# Supplementary material for: Molecular Cloning, Screening of Single Nucleotide Polymorphisms, and Analysis of Growth-Associated Traits of igf2 in Spotted Sea Bass (Lateolabrax maculatus)
Source: Animals (Basel). 2023 Mar 8;13(6):982. doi: 10.3390/ani13060982 (PMC10044540; doi:10.3390/ani13060982)
Supplement: Supplementary file 1 [file animals-13-00982-s001.zip › animals-2068853-supplementary.pdf]

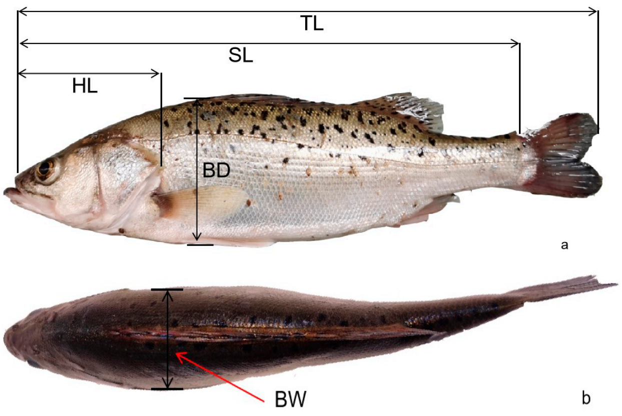

Figure S1. Measurement of *L. maculatus* body in this study. a: side view, b: dorsal view. TL: total length, SL: standard length, HL: head length, BD: body depth, BW: body width.
